# Association of health‐care and long‐term care resources for patients with dementia with institutionalization and death: Evidence from South Korea

**DOI:** 10.1002/alz.70271

**Published:** 2025-05-12

**Authors:** Sujin Kim, Jaeeon Yoo

**Affiliations:** ^1^ Department of Health Care Policy Research Korea Institute for Health and Social Affairs Sejong‐si Republic of Korea; ^2^ Department of Social Welfare Gachon University Seongnam‐si Republic of Korea

**Keywords:** community‐based care, health‐care workforce, home‐care workforce, institutionalization, long‐term care facilities, mortality, regional disparities

## Abstract

**INTRODUCTION:**

The role of community characteristics on supporting the community residence of older adults with dementia has received little attention. This study explores the association of community resources with extending older adults’ stay in community settings and mitigating premature institutionalization and mortality.

**METHODS:**

Using data from 286,940 patients with dementia in South Korea, this study used Cox proportional hazard models to analyze the relationships among community management, institutionalization, mortality rates, and the availability of long‐term care and medical resources at the municipal level, adjusting for individual factors.

**RESULTS:**

Increased availability of home care nurses and physicians was significantly associated with enhanced community management, showing a noticeable decrease in mortality and institutionalization risk. Additionally, more home care social workers were associated with a reduced institutionalization risk, while an excess of medical and long‐term care facilities was linked to a heightened institutionalization risk.

**DISCUSSION:**

These findings underscore the importance of health‐care resources aligning with the specific needs of patients with dementia.

**Highlights:**

- Local health‐care resources may affect dementia patients’ community residence.
- Results reveal physicians and home case nurses lower institutionalization and death.
- Study findings indicate home care social workers also reduce institutionalization.
- But, more hospital beds and long‐term care facilities increase institutionalization.
- This underscores the need for nuanced approaches to community‐based care.

## 1 INTRODUCTION

Residing in one's local community during the later stages of life is a pivotal element of self‐determination and the dignified conclusion of one's life. In the United States, > 85% of individuals aged ≥ 65 years express a preference for aging in their current residence or community.^1^ According to the 2020 Older Adult Survey conducted among 10,000 individuals aged ≥ 65 years in South Korea, 70% preferred to continue living in their homes and communities despite declining health, rather than opting for long‐term care (LTC) facilities.^2^ However, providing sufficient care within homes or local communities for older adults with dementia poses considerable challenges, often leading them to seek admission to LTC facilities or medical hospitals where they eventually face the end of life.^3^, ^4^, ^5^

Prolonged residency in LTC facilities or admission to medical hospitals not only represents an undesirable choice for individuals affected by dementia but also triggers increased societal expenditure on long‐term care insurance (LTCI) and health insurance.^6^, ^7^ In South Korea, for older adults classified as Level 3 under the Long‐Term Care Insurance system, the cost of institutional care services is > 1.6 times higher than the maximum allowable amount for home care services.^8^ Older adults with dementia who reside long term in nursing hospitals or care facilities often face isolation from the outside world, resulting in decreased physical activity, diminished social engagement, and reduced interpersonal interactions, along with increased feelings of loneliness and depression.^9^ According to the World Health Organization, > 55 million cases of dementia exist worldwide, with nearly 10 million new cases emerging yearly.^10^ South Korea also shows an annual increase in 50,000 patients with dementia, and the annual management cost per dementia patient constitutes ≈ 50% of the annual household income.^11^ With the rapidly aging population, this burden is expected to become even more severe. In this context, understanding the factors influencing the choices of older adults with dementia between residence continuity and community living is imperative. To delay institutionalization and end‐of‐life care for patients with dementia, thereby extending community residence, this study aims to elucidate the influencing factors.

Various studies have extensively explored the factors that contribute to prolonged institutionalization or premature mortality in patients with dementia.^4^, ^12^, ^13^, ^14^, ^15^, ^16^ It has been established that the risk of institutionalization or death increases with factors such as advanced age,^12^, ^13^, ^14^ female sex,^4^, ^15^ low socioeconomic status,^4^, ^15^, ^16^ severe disability,^13^, ^14^, ^16^ and the presence of multiple comorbidities.^4^, ^13^, ^14^ In particular, older adults with dementia constitute > 50% of residents in LTC facilities and are significantly more likely to undergo institutionalization compared to those without dementia.^17^ Furthermore, they often experience adverse health outcomes, including a higher prevalence of falls and an elevated risk of delirium after transitions to nursing homes.^18^ Extensive research exists on the individual characteristics that affect patients with dementia. However, there remains a notable knowledge gap regarding the influence of community attributes—particularly those concerning LTC, medical facilities, and workforce supply levels—on community management. Identifying the challenges that necessitate policy intervention is crucial.

**RESEARCH IN CONTEXT**

- **Systematic review**: The authors conducted a comprehensive search across academic databases (e.g., PubMed). While numerous studies have examined individual risk factors for nursing home admission, few have investigated the association of community health‐care and long‐term care resources for patients with dementia with institutionalization and mortality rate.
- **Interpretation**: This study thoroughly examines the factors affecting housing choices and challenges for older adults with dementia, highlighting community health‐care and home‐care resources. Its insights are crucial for devising strategies to delay institutionalization and reduce premature mortality, underscoring the importance of informed policies amid the rising global burden of dementia.
- **Future directions**: Future research could explore the following areas: (1) conducting studies that broaden the geographical scope to encompass diverse countries for comparative analysis, and (2) developing strategies to enhance health‐care policies aimed at improving the effectiveness of health‐care resources within local communities.

Attention has been directed toward regional imbalances in the proportion of older adult individuals living alone, urbanization rates, and disparities in the supply of resources such as LTC facilities, medical hospitals, social workers, care aides, nurses, and physicians.^19^, ^20^, ^21^, ^22^ Despite a few studies using cross‐sectional data and multilevel models to analyze the emotional well‐being, loneliness, and community satisfaction of older adults based on facility and workforce supply levels, there remains a noticeable scarcity of research in this area, including at the international level.^19^, ^20^, ^21^ Additionally, research analyzing longitudinal data to understand how regional environmental factors contribute to community management, institutionalization, and death in patients with dementia is lacking.^14^, ^15^

This study emphasizes the need for comprehensive research to bridge the existing gap in understanding how regional characteristics, including facility and workforce supply levels, influence the progression to community management, institutionalization, and death in patients with dementia. Using large data from the National Health Insurance Service (NHIS) database in South Korea, this study focuses on patients newly diagnosed with dementia aged ≥ 45 years between 2013 and 2021, excluding those with a history of dementia, using multivariate Cox proportional hazard regression models. These analyses are expected to provide valuable insights into the complex interplay of individual and environmental factors, informing the development of more effective community care strategies. Such strategies aim to delay institutionalization and mortality, while also ensuring care aligns with the preferences of individuals with dementia to remain within their communities and reduce LTC expenditures.

## 2 METHODS

### 2.1 Data sources

The study population data were obtained from the NHIS database of South Korea. The NHIS provides mandatory health‐care coverage for the entire 50 million population of South Korea, including National Health Insurance (NHI) enrollees (97%), Medical Aid beneficiaries (3%), and finances and management of LTC services. Public LTCI was introduced in 2008. Eligibility for LTC services is determined based on a needs assessment for individuals aged ≥ 65 years, as well as those < 65 years with geriatric diseases. The NHIS covers most forms of health services, including emergency, inpatient, and outpatient care, as well as medication prescriptions and formal LTC services such as facilities and home‐based care. The NHIS database contains records of all demographics, including residence, clinical information on inpatient and outpatient visits, procedures, prescriptions, and diagnosis records (coded using the International Classification of Diseases, 10th Revision [ICD‐10]), and LTCI service use information.^23^ In addition, this database contains death records obtained from data linked to South Korea's national statistics. A sample of 10,413,089 participants, constituting 20% of the 2012 population, was drawn from the NHIS database, ensuring representation across sex, age, and region distributions. Participants were followed up until December 31, 2021, with their insurance claims of medical institutions and enrollee information collected for analysis (data number: NHIS‐2022‐1‐629). Information on community health‐care resources was obtained from the National Statistical Information on Service.

### 2.2 Study population

We defined the study population as individuals newly diagnosed with dementia, aged ≥ 45, between 2013 and 2021. Newly diagnosed patients had used inpatient care or had more than two outpatient visits without a history of dementia diagnostic codes 1 year prior, according to ICD‐10 codes (F00, F01, F02, F03, G30, G3100, G3101, G3102, G3103, G3104, and G3182).^24^ Among the 386,465 newly diagnosed patients with dementia, we excluded 92,326 who had been admitted to medical hospitals or LTC facilities for > 3 months at the time of diagnosis, which was considered institutionalization, as discussed later. After excluding 7199 patients with missing covariates, 286,940 patients were analyzed. All patients were followed‐up from their diagnosis date until the occurrence of the outcome or the end of the study period (December 31, 2021), whichever occurred first.

### 2.3 Outcomes

Our primary outcome measure was “community management,” which refers to the period during which individuals with dementia are managed and cared for in their own homes or community settings, rather than in institutional facilities. The duration of community management was assessed from the time of dementia diagnosis to the endpoint, which was defined as the patient's passing or their admission to a nursing facility or medical institution for > 3 months, based on previous literature.^25^, ^26^ Recognizing that individuals ineligible for LTC care or with other severe conditions may be admitted to hospitals instead of nursing homes, long‐term hospitalization was considered a form of institutionalization. In addition, we conducted separate analyses of institutionalization in nursing facilities or hospitals and mortality.

### 2.4 Socio‐demographic and clinical characteristics

Sex, health coverage type, income, disability severity, and comorbidities were also included as covariates. Health‐care coverage included NHI enrollees and Medical Aid, a subsidy program for people experiencing poverty. Income level was categorized into five groups using NHI contribution quintiles: Medical Aid and the first, second, third, fourth, and fifth contribution quintiles. Disability severity was categorized into three groups—no disability, moderate disability, and severe disability—based on a national disability registration system established by the Ministry of Health and Welfare to provide appropriate welfare benefits.^27^

This study included comorbidities as time‐varying confounders because they may not only change after the diagnosis of dementia but also affect community management. Comorbidities were measured every 6 months using the Charlson Comorbidity Index (CCI), which reflects the number and seriousness of pre‐existing comorbid conditions and was calculated as the sum of a weighted index of 1 to 6 points for 17 disease groups,^28^, ^29^ including myocardial infarction, congestive heart failure, peripheral vascular disease, cerebrovascular disease, dementia, chronic pulmonary disease, rheumatologic disease, peptic ulcer disease, liver disease, diabetes, hemiplegia or paraplegia, renal disease, malignancy, and acquired immunodeficiency.

### 2.5 Municipality characteristics

South Korea has a three‐tiered administrative system comprising 17 first‐tier administrative divisions, which are further subdivided into a variety of smaller entities: municipalities, including cities, counties, and districts. Our final dataset included 251 municipalities out of a total of 253 municipalities. Factors related to regional health‐care resources include the number of doctors and beds per 10,000 individuals at municipal level. Based on the results of previous studies, we included the proportion of older adult households where adults aged ≥ 65 years live alone per 1000 households and the proportion of urban residents per 10 of the total population into the analysis.

The LTCI in South Korea encompasses institutional care, such as nursing homes, and home‐based care. It determines an individual's eligibility for LTC services and the level of care using a standardized assessment tool. Initially, the LTC system consisted of three levels, which were expanded to five levels in 2014 to include patients with dementia without severe physical limitations.^30^ Subsequently, in 2018, it was further expanded to six levels, including individuals with mild cognitive impairment but without physical functional limitations.^30^ However, facility‐based services were available only to individuals with higher levels of care needs, such as those classified in levels 1, 2, and 3, who experienced significant functional limitations. Home‐based care includes home‐visit care, home‐visit bathing, home‐visit nursing, adult day and overnight stay care, and assistive devices.^30^ These services are provided by nurses, social workers, and care workers. Hence, we measured regional LTC resources using the number of beds in LTC facilities for institutional care and the number of social workers, nurses, and care workers for home‐based care per 100 persons aged ≥ 65 years in each municipality to identify the effects of community LTC resources.

### 2.6 Statistical analyses

Multivariate Cox proportional hazard regression analyses were conducted to evaluate the adjusted hazard ratios (aHRs) and 95% confidence intervals (CIs) for outcomes using age as the time scale. Attained age, defined on a day‐unit basis, was based on the study participants’ ages at the time of diagnosis and at the occurrence of an event or the censoring of their follow‐up. All Cox models were stratified by sex, residence, disability level, income, health coverage type, and diagnosis year due to violations of the proportional hazard assumption. The variables of regional LTC resources, urbanization, and the proportion of older adults living alone were included in all multivariate models with time‐varying CCI. The proportional hazard assumption was assessed by including time‐dependent covariates, which were created from interactions between the predictors and survival time in Cox proportional hazard regression models.^31^ Data analyses were performed using SAS Enterprise Guide 7.1 (SAS Institute).

### 2.7 Ethics

As this study used de‐identified data provided by the NHIS after anonymization according to strict confidentiality guidelines, the ethics review was exempted by the institutional review board (RB) of Korea Institute for Health and Social Affairs (IRB number: 2022‐004).

## 3 RESULTS

### 3.1 Characteristics of the study population

A total of 286,940 eligible patients were included in this study, comprising 181,141 female and 105,799 male patients. The average age was 77.3 years, with a standard deviation of 9.4 years. Table 1, and Table S1 and Figure S1 in supporting information present the baseline characteristics categorized by community management status, comparing censored cases (community management) to those experiencing events such as death or institutionalization. Incomes in the lowest quintile were more prevalent in the event group than in the censored group (28.3% and 24.8%, respectively). Most participants had no disabilities, and the proportion of those with disabilities was higher in the event group (moderate, 16.2%; severe, 9.4%) than in the censored group (moderate, 15.5%; severe, 5.8%). CCI scores tended to be higher in the event group, with only 34.7% displaying a score of 0, compared to 37.6% in the censored group. The proportions of older adult households and urbanization were higher in the censored group. In addition, the censored group exhibited a higher number of home‐visit social workers, nurses, and care workers. The number of institutional care beds in LTC facilities and hospital beds was higher in the censored group, with marginal statistical significance. Table S2 in supporting information presents the correlation matrix among all variables used in the analysis.

**TABLE 1:** Descriptive statistics.

	Total *N* = 286,940	Censored *n* = 150,707	Event occurred^*^ *n* = 136,233	*p* value
Female, *n* (%)	181,141 (63.13)	100,074 (66.40)	81,067 (59.51)	<0.001
Male, *n* (%)	105,799 (36.87)	50,633 (33.60)	55,166 (40.49)	
Age, mean (SD), y	77.26 (9.35)	75.49 (9.33)	80.21 (8.45)	<0.001
National health insurance, *n* (%)	251,571 (87.67)	134,025 (88.93)	117,546 (86.28)	<0.001
Medical Aid, *n* (%)	35,369 (12.33)	16,682 (11.07)	18,687 (13.72)	
Lowest quintile income, *n* (%)	75,965 (26.47)	37,353 (24.79)	38,612 (28.34)	<0.001
2nd quintile income, *n* (%)	26,848 (9.36)	14,270 (9.47)	12,578 (9.23)	
3rd quintile income, *n* (%)	35,674 (12.43)	19,007 (12.61)	16,667 (12.23)	
4th quintile income, *n* (%)	50,421 (17.57)	27,350 (18.15)	23,071 (16.93)	
Highest quintile income, *n* (%)	98,032 (34.16)	52,727 (34.99)	45,305 (33.26)	
No disability, *n* (%)	219,982 (76.66)	118,551 (78.66)	101,431 (74.45)	<0.001
Moderate disability, *n* (%)	45,406 (15.82)	23,369 (15.51)	22,037 (16.18)	
Severe disability, *n* (%)	21,552 (7.51)	8787 (5.83)	12,765 (9.37)	
CCI = 0, *n* (%)	104,006 (36.25)	56,682 (37.61)	47,324 (34.74)	<0.001
CCI = 1, *n* (%)	80,618 (28.10)	44,015 (29.21)	36,603 (26.87)	
CCI = 2, *n* (%)	55,681 (19.41)	28,357 (18.82)	27,324 (20.06)	
CCI ≥ 3, *n* (%)	46,635 (16.25)	21,653 (14.37)	24,982 (18.34)	
Living alone^†^	0.895 (0.52)	0.905 (0.51)	0.884 (0.53)	<0.001
Urbanization^†^	8.348 (2.28)	8.421 (2.24)	8.268 (2.33)	<0.001
Number of LTC facility beds^‡^	2.431 (1.32)	2.436 (1.37)	2.426 (1.26)	0.058
Number of home‐visit social workers^‡^	0.184 (0.09)	0.210 (0.09)	0.156 (0.09)	<0.001
Number of home‐visit nurses^‡^	0.020 (0.02)	0.021 (0.02)	0.019 (0.02)	<0.001
Number of home‐visit care workers^‡^	4.732 (1.84)	5.001 (1.86)	4.434 (1.76)	<0.001
Number of doctors^§^	0.273 (0.19)	0.280 (0.19)	0.265 (0.19)	<0.001
Number of hospital beds^§^	1.471 (0.83)	1.474 (0.83)	1.467 (0.83)	0.055

Abbreviations: CCI, Charlson Comorbidity Index; LTC, long‐term care; SD, standard deviation.

* Event was defined as death or institutionalization.

^†^ Older adult households where adults aged ≥ 65 years live alone per 1000 households and the proportion of urban residents per 10 of the total population in each municipality.

^‡^ The number of beds in LTC facilities for institutional care and the number of social workers, nurses, and care workers for home‐based care per 100 persons aged ≥ 65 years in each municipality.

^§^ The number of doctors and beds per 10,000 persons in each municipality.

### 3.2 Association of LTC resources with death or institutionalization

Table 2 and Table S3 in supporting information present the results of the Cox regression analysis. Crude HRs indicated correlations between several factors and the likelihood of death or institutionalization. Higher CCI scores, a greater proportion of older adult households, greater urbanization, a higher number of LTC facility beds, a greater number of home‐based social workers and care workers, and a higher number of hospital beds were associated with a higher likelihood of death or institutionalization. Conversely, a higher number of home‐care nurses and doctors was correlated with a lower likelihood of death or institutionalization.

**TABLE 2:** Cox proportional hazards regression analysis results: Death or institutionalization.

	Crude ratios		Adjusted ratios	
	HR (95% CI)	*p* value	HR (95% CI)	*p* value
CCI = 1 (ref. CCI = 0)	1.051 (1.031, 1.072)	<0.001	1.024 (1.002, 1.047)	0.036
CCI = 2	0.987 (0.970, 1.003)	0.118	1.001 (0.982, 1.020)	0.937
CCI ≥ 3	1.180 (1.163, 1.199)	<0.001	1.162 (1.142, 1.183)	<0.001
Living alone	1.031 (1.021, 1.042)	<0.001	1.006 (0.978, 1.033)	0.694
Urbanization	1.000 (0.997, 1.002)	0.682	1.011 (1.004, 1.018)	0.001
LTC facility beds	1.018 (1.014, 1.023)	<0.001	1.020 (1.013, 1.028)	<0.001
Home‐visit social workers	1.559 (1.464, 1.661)	<0.001	0.795 (0.663, 0.955)	0.014
Home‐visit nurses	0.434 (0.317, 0.594)	<0.001	0.606 (0.401, 0.918)	0.018
Home‐visit care workers	1.006 (1.003, 1.009)	<0.001	0.994 (0.987, 1.000)	0.058
Doctors	0.927 (0.901, 0.954)	<0.001	0.932 (0.894, 0.971)	0.001
Hospital beds	1.029 (1.023, 1.035)	<0.001	1.016 (1.005, 1.027)	0.004

Abbreviations: CCI, Charlson Comorbidity Index; CI, confidence interval; HR, hazard ratio; LTC, long‐term care.

Adjusted: Estimation from multivariate Cox proportional hazard regression analyses for outcomes, using age as the time scale and stratified by sex, residence, disability level, income, health coverage type, and diagnosis year.

Living alone: The proportion of older adult households where adults aged ≥ 65 years live alone, per 1000 households.

Urbanization: The proportion of urban residents per 10 residents in each municipality.

LTC facility beds: The number of beds in LTC facilities (for institutional care) per 100 persons aged ≥ 65 years.

Home‐visit social workers, home‐visit nurses, and home‐visit care workers: The number of social workers, nurses, and care workers for home‐based care per 100 persons aged ≥ 65 years.

Doctors and hospital beds: The number of doctors and hospital beds per 10,000 persons in each municipality.

After adjusting for covariates, some associations displayed differences. A higher ratio of LTC facility capacity per older adult population and more medical hospital beds per population were correlated with a higher likelihood of death or institutionalization, with aHRs of 1.020 (95% CI: 1.013–1.028) and 1.016 (95% CI: 1.005–1.027), respectively. A higher ratio of home care social workers and nurses per older adult population was negatively associated with death or institutionalization, with aHRs of 0.795 (95% CI: 0.663–0.955) and 0.606 (95% CI: 0.401–0.918), respectively. Additionally, the number of care workers was correlated with death or institutionalization, with marginal statistical significance (*p* value = 0.058). A higher ratio of doctors per population was also negatively associated with death or institutionalization, with an aHR of 0.932 (95% CI: 0.894–0.971). Additionally, higher CCI scores and residing in areas with greater urbanization were associated with higher rates of death or institutionalization compared to their counterparts.

### 3.3 Association of LTC resources with death

We analyzed the risk factors for death (Table 3 and Table S3). A higher ratio of nurses and care workers was associated with a reduced likelihood of death, showing aHRs of 0.584 (95% CI: 0.367–0.928) and 0.992 (95% CI: 0.985–1.000), respectively. In addition, a higher ratio of doctors reduced death with an aHR of 0.924 (95% CI: 0.882, 0.968).

**TABLE 3:** Cox proportional hazards regression analysis results: Death.

	Crude ratios		Adjusted ratios	
	HR (95% CI)	*p* value	HR (95% CI)	*p* value
CCI = 1 (ref. CCI = 0)	1.170 (1.145, 1.196)	<0.001	1.166 (1.132, 1.202)	<0.001
CCI = 2	0.833 (0.817, 0.849)	<0.001	1.241 (1.211, 1.271)	<0.001
CCI ≥ 3	0.986 (0.970, 1.003)	0.113	1.702 (1.664, 1.740)	<0.001
Older adult household	1.025 (1.013, 1.036)	<0.001	1.009 (0.978, 1.040)	0.586
Urbanization	1.001 (0.998, 1.003)	0.639	1.005 (0.997, 1.012)	0.205
Number of LTC facility beds	1.008 (1.003, 1.013)	0.002	1.005 (0.997, 1.013)	0.211
Number of home‐visit social workers	2.027 (1.885, 2.179)	<0.001	0.961 (0.782, 1.182)	0.706
Number of home‐visit nurses	0.758 (0.533, 1.078)	0.123	0.584 (0.367, 0.928)	0.023
Number of home‐visit care workers	1.013 (1.009, 1.016)	<0.001	0.992 (0.985, 1.000)	0.041
Number of doctors	0.953 (0.923, 0.983)	0.003	0.924 (0.882, 0.968)	0.001
Number of hospital beds	1.011 (1.004, 1.019)	0.002	1.010 (0.998, 1.022)	0.117

Abbreviations: CCI, Charlson Comorbidity Index; CI, confidence interval; HR, hazard ratio; LTC, long‐term care.

Adjusted: Estimation from multivariate Cox proportional hazard regression analyses for outcomes, using age as the time scale and stratified by sex, residence, disability level, income, health coverage type, and diagnosis year.

Living alone: The proportion of older adult households where adults aged ≥ 65 years live alone, per 1000 households.

Urbanization: The proportion of urban residents per 10 residents in each municipality.

LTC facility beds: The number of beds in LTC facilities (for institutional care) per 100 persons aged ≥ 65 years.

Home‐visit social workers, home‐visit nurses, and home‐visit care workers: The number of social workers, nurses, and care workers for home‐based care per 100 persons aged ≥ 65 years.

Doctors and hospital beds: The number of doctors and hospital beds per 10,000 persons in each municipality.

### 3.4 Association of LTC resources with institutionalization

Institutionalization decreased with higher ratios of home care social workers and nurses, showing aHRs of 0.680 (95% CI: 0.531–0.870) and 0.550 (95% CI: 0.315–0.961), respectively (Table 4 and Table S3). The number of doctors also had a negative association with institutionalization, with an aHR of 0.917 (95% CI: 0.867–0.970). In contrast, higher ratios of LTC facility capacity and medical hospitals were related to an increased likelihood of institutionalization, showing aHRs of 1.032 (95% CI: 1.022–1.042) and 1.019 aHR (95% CI: 1.005–1.034), respectively.

**TABLE 4:** Cox proportional hazards regression analysis results: Institutionalizations.

	Crude ratios		Adjusted ratios	
	HR (95% CI)	*p* value	HR (95% CI)	*p* value
CCI = 1 (ref. CCI = 0)	0.962 (0.936, 0.987)	0.004	0.932 (0.905, 0.960)	<0.001
CCI = 2	0.769 (0.752, 0.786)	<0.001	0.985 (0.961, 1.010)	0.230
CCI ≥ 3	0.738 (0.723, 0.753)	<0.001	1.047 (1.023, 1.071)	<0.001
Older adult household	1.019 (1.005, 1.033)	0.007	0.999 (0.963, 1.036)	0.965
Urbanization	0.998 (0.995, 1.001)	0.267	1.015 (1.006, 1.024)	0.001
Number of LTC facility beds	1.025 (1.019, 1.030)	<0.001	1.032 (1.022, 1.042)	<0.001
Number of home‐visit social workers	0.695 (0.637, 0.759)	<0.001	0.680 (0.531, 0.870)	0.002
Number of home‐visit nurses	0.173 (0.112, 0.267)	<0.001	0.550 (0.315, 0.961)	0.036
Number of home‐visit care workers	0.988 (0.984, 0.992)	<0.001	0.996 (0.987, 1.005)	0.350
Number of doctors	0.867 (0.833, 0.902)	<0.001	0.917 (0.867, 0.970)	0.002
Number of hospital beds	1.039 (1.030, 1.048)	<0.001	1.019 (1.005, 1.034)	0.009

Abbreviations: CCI, Charlson Comorbidity Index; CI, confidence interval; HR, hazard ratio; LTC, long‐term care.

Adjusted: Estimation from multivariate Cox proportional hazard regression analyses for outcomes, using age as the time scale and stratified by sex, residence, disability level, income, health coverage type, and diagnosis year.

Living alone: The proportion of older adult households where adults aged ≥ 65 years live alone, per 1000 households.

Urbanization: The proportion of urban residents per 10 residents in each municipality.

LTC facility beds: The number of beds in LTC facilities (for institutional care) per 100 persons aged ≥ 65 years.

Home‐visit social workers, home‐visit nurses, and home‐visit care workers: The number of social workers, nurses, and care workers for home‐based care per 100 persons aged ≥ 65 years.

Doctors and hospital beds: The number of doctors and hospital beds per 10,000 persons in each municipality.

## 4 DISCUSSION

This study analyzed the relationship between the potential for community management and the level of LTC and medical resources at the municipal level using Cox regression analysis of data. Results indicated that the supply of LTC facilities and medical institution beds within the community was generally associated with reduced community management, particularly through an increase in institutionalization. Conversely, an increase in the supply of medical and caregiving providers, such as physicians and nurses for home‐based care within the community, was generally associated with enhanced community management and a lower occurrence of death or institutionalization. Further, the presence of community social workers was associated with improved community management and a reduced risk of institutionalization. In addition, consistent with previous studies, clinical characteristics such as CCI were found to be relevant to community management, death, and institutionalization as in our study while urbanization was associated with community management and institutionalization.

### 4.1 Relationship between the health‐care workforce and community management

Our study found that a higher supply of home care nurses and doctors was associated with improved community management of patients with dementia, along with notable reductions in both mortality and institutionalization rates. Previous literature has identified a positive association between medical doctors, especially primary care physicians, and health outcomes.^32^, ^33^, ^34^ This literature has suggested that the benefits of the primary care workforce are derived from the amount of ambulatory clinical care provided rather than the number of locally available primary care physicians. Early detection, prevention, and care coordination may prevent health deterioration and reduce hospital stay.^32^ Notably, South Korea has 2.6 physicians per 1000 persons, a figure relatively low compared to countries of similar economic status, and there are concerns about the unequal distribution of health‐care resources.^35^ Our findings may be particularly relevant in the context of this shortage of physicians and unequal distribution, highlighting the potential importance of the number of doctors for older adults to remain in the community. Further studies are warranted to corroborate these findings.

Additionally, although several systematic reviews have not observed a clear relationship between nurse staffing and health outcomes in long‐term institutional care,^36^, ^37^ recent literature emphasized the positive role of nurses in nursing‐sensitive outcomes such as activities of daily living, falls, infection, pain, and weight loss.^38^ Although research on nurse staffing in home‐visit LTC remains limited, a study conducted in the United States found an association between home health‐care services provided by registered nurses and improvements in patients’ quality of life and overall health.^39^ Additionally, prior evidence suggests an association between home‐visit nursing care and a lower likelihood of nursing home admissions, potentially through mitigating cognitive and functional decline.^40^, ^41^ In South Korea, where home care nurses predominantly support older adults with physical limitations, increasing the proportion of home care nurses may be associated with a reduced risk of institutionalization and better outcomes for individuals living with dementia.

A higher supply of social workers for home‐based care was correlated with lower institutionalization rates among patients with dementia (higher likelihood of community management). Previous literature has suggested that social workers play a role in assessing patients’ psychosocial needs, developing collaborative treatment plans, and connecting patients to available resources in the community, which may support patients in remaining safe and independent in their homes.^42^ Accordingly, patients with dementia may have benefited from continuing to live in the community, particularly in areas with a greater number of social workers providing home care. However, the lack of strong statistical significance in mortality observed in our study suggests that the role of social workers alone may be limited for patients with dementia who require medical services. Consequently, there has been growing advocacy for integrated services that combine home nursing care provided by nurses with social work support. This perspective aligns with the associations observed in our study's findings.

### 4.2 Association of hospital beds and LTC facilities with community management

The findings regarding hospital beds and LTC facilities within the local community suggest that the availability of care and medical facilities within the community may be associated with reduced likelihood of continued residence for patients with dementia in the community and higher rates of institutionalization, highlighting certain concerning patterns. Theoretically, an increase in the supply of LTC facilities or hospitals to an appropriate level within the community may improve service accessibility and extend lifespan compared to areas with inadequate supply. Globally, previous studies have emphasized associations between health‐care resource availability and patient outcomes, including institutionalization and mortality.^14^, ^21^, ^43^ However, exceeding a certain threshold in such provisions may be linked to inter‐provider competition and unnecessary institutionalization. Particularly noteworthy is South Korea's high number of hospital beds compared to other Organisation for Economic Co‐operation and Development countries.^35^ This heightened availability and competition among medical facilities may be associated with an increased likelihood of patients with dementia being unnecessarily institutionalized or experiencing premature mortality, aligning with the notion that an oversupply of medical services may steer individuals toward institutional settings rather than community‐based care.

Although empirical findings from Korea are limited, some qualitative studies have raised concerns regarding such occurrences.^44^ Our research supports these arguments using empirical evidence. This association highlights the importance of balancing and appropriately allocating medical resources within communities. Therefore, a more nuanced approach to health‐care planning is necessary to ensure that the supply of medical resources aligns with the actual needs of the population, particularly considering the unique care requirements of patients with dementia.

This study also highlights the relevance of reassessing and enhancing community‐based care strategies to better support patients with dementia and their families. The findings on the higher probability of institutionalization with a greater ratio of medical hospital beds per population provide valuable insights into the potential consequences of health‐care resource distribution. The association between higher facility ratios and increased likelihood of institutionalization raises questions about the adequacy of community‐based care and its ability to meet the diverse needs of patients with dementia in their localities. Consequently, these identified associations underscore the need for a comprehensive examination of community‐based care models for patients with dementia. Both academics and caregiving practitioners should consider these findings in ongoing discussions on dementia care, foster discourse on policy implications, and develop community‐based interventions tailored to the specific needs of patients with dementia to enhance their well‐being within local communities.

### 4.3 Association of CCI and urbanization level with community management

Although not the primary independent variables, consistent findings suggest that high numbers of comorbidities and increased urbanization levels are associated with challenges in the community management of patients with dementia. While dementia is a critical factor linked to institutionalization, comorbidities may also be associated with institutionalization and mortality in persons with dementia.^45^, ^46^, ^47^ In addition, one study suggested that the higher likelihood of admission to long‐term institutional care in urban residents may be related to a different attitude toward institutional care compared to rural residents.^40^ It may also be related to the less available family home caregiver in urban environments. Future studies could explore more nuanced examinations, including investigating the impact of comorbidities and urbanization. In addition, the development of medical and caregiving interventions aimed at alleviating the adverse effects of comorbidities and urbanization on the community management of patients with dementia should be advanced.

### 4.4 Limitations

This study has some limitations. First, while household characteristics such as living arrangements may impact community management rates, this information was not available. To mitigate this limitation, we included the municipal‐level proportion of older adult individuals living alone in our analysis. Second, the operationalization of the local community, as defined through aggregated and publicly disclosed municipality‐level formal statistics, may introduce a spatial mismatch concerning the geographic extent of care and medical services received by patients with dementia. Thus, subsequent research is encouraged to investigate the spatial scope using diverse approaches, such as those derived from administrative statistics at the municipal level, which may diverge from those generated by official statistics. Third, beyond the second‐level variables incorporated in this study, a more comprehensive examination of community characteristics that can influence the institutionalization or mortality of patients with dementia is imperative. Regional variations in education level, caregiver burden, and functional limitations, for instance, may influence institutionalization and mortality. However, these variables were not accessible for inclusion in this study, which was based on secondary data analysis. Fourth, the analysis results of this study indicate associations rather than strict causal relationships, and the possibility of reverse causality between variables cannot be excluded. Future research should explore the potential reverse causality where the demand for LTC facilities and nursing hospitals within the community may drive their supply.

## 5 CONCLUSIONS

This study has academic and practical significance, delving into factors influencing housing choices for older adults with dementia and addressing challenges of prolonged institutionalization and premature mortality. It addresses a notable gap in existing literature by highlighting the often‐overlooked influence of community characteristics such as LTC facilities and workforce supply levels. The use of longitudinal data distinguishes this research, offering insights into how regional environmental factors contribute to the progression of institutionalization or death over time. Findings suggested that prioritizing replenishing physicians, social workers, and nurses providing home‐based care rather than simply increasing medical and LTC facilities to promote the continued residence of patients with dementia in the local community. These insights are pertinent for health‐care practitioners, policy makers, and researchers striving for supportive and sustainable community environments for older adults with dementia.

## CONFLICT OF INTEREST STATEMENT

The authors declare that they have no known competing financial interests or personal relationships that could have appeared to influence the work reported in this paper. Author disclosures are available in the supporting information.

## CONSENT STATEMENT

We confirm that consent was not required because this study analyzed secondary data collected and provided by the National Health Insurance Service.

## Supporting information



Supporting Information

Supporting Information
