# Supplementary material for: Association of health‐care and long‐term care resources for patients with dementia with institutionalization and death: Evidence from South Korea
Source: Alzheimers Dement. 2025 May 12;21(5):e70271. doi: 10.1002/alz.70271 (PMC12069021; doi:10.1002/alz.70271)
Supplement: Supplementary file 1 — Supporting Information [file ALZ-21-e70271-s001.docx]

Supplementary Tables and Figures

Table S1. Descriptive Statistics

|  | | Total  N=286,940 | Censored  n = 150,707 | Event occurred^*^  n = 136,233 | *p* value |
| --- | --- | --- | --- | --- | --- |
| Diabetes | No, n (%) | 173,123 (60.33) | 91,645 (60.81) | 81,478 (59.81) | <0.001 |
|  | Yes, n (%) | 113,817 (39.67) | 59,062 (39.19) | 54,755 (40.19) |  |
| Congestive Heart Failure | No, n (%) | 246,641 (85.96) | 132,841 (88.15) | 113,800 (83.53) | <0.001 |
|  | Yes, n (%) | 40,299 (14.04) | 17,866 (11.85) | 22,433 (16.47) |  |
| Stroke | No, n (%) | 241,251 (84.08) | 128,278 (85.12) | 112,973 (82.93) | <0.001 |
|  | Yes, n (%) | 45,689 (15.92) | 22,429 (14.88) | 23,260 (17.07) |  |
| Myocardial infarction | No, n (%) | 277,810 (96.82) | 146,768 (97.39) | 131,042 (96.19) | <0.001 |
|  | Yes, n (%) | 9,130 (3.18) | 3,939 (2.61) | 5,191 (3.81) |  |
| Cancer | No, n (%) | 256,317 (89.33) | 137,590 (91.30) | 118,727 (87.15) | <0.001 |
|  | Yes, n (%) | 30,623 (10.67) | 13,117 (8.70) | 17,506 (12.85) |  |
| Renal diseases | No, n (%) | 285,778 (99.60) | 150,314 (99.74) | 135,464 (99.44) | <0.001 |
|  | Yes, n (%) | 1,162 (0.40) | 393 (0.26) | 769 (0.56) |  |

CCI: Charlson Comorbidity Index. LTC: Long-Term-Care.

^*^Event was defined as death or institutionalization.

Table S2. Correlation matrix displaying relationship between all variables

|  | **1** | **2** | **3** | **4** | **5** | **6** | **7** | **8** | **9** | **10** | **11** | **12** | **13** | **14** | **15** | **16** | **17** | **18** | **19** | **20** |
| --- | --- | --- | --- | --- | --- | --- | --- | --- | --- | --- | --- | --- | --- | --- | --- | --- | --- | --- | --- | --- |
| **1.Disability** | 1.000 | -0.034 | 0.071 | 0.003 | 0.027 | 0.049 | 0.043 | 0.034 | 0.065 | 0.016 | 0.014 | 0.022 | 0.009 | 0.023 | 0.001 | 0.000 | 0.021 | -0.015 | -0.013 | 0.013 |
| **2.Income** | -0.034 | 1.000 | -0.516 | 0.003 | 0.001 | -0.015 | -0.017 | -0.013 | -0.004 | -0.008 | 0.018 | 0.001 | -0.026 | -0.005 | 0.008 | 0.003 | -0.026 | 0.010 | 0.011 | -0.015 |
| **3.Medical Aid** | 0.071 | -0.516 | 1.000 | 0.001 | 0.013 | 0.042 | 0.040 | 0.027 | 0.024 | 0.015 | -0.003 | 0.002 | -0.003 | -0.002 | 0.002 | 0.009 | 0.001 | 0.019 | 0.007 | 0.026 |
| **4.CCI=1** | 0.003 | 0.003 | 0.001 | 1.000 | -0.307 | -0.275 | 0.095 | -0.061 | 0.070 | -0.067 | -0.033 | -0.023 | -0.003 | -0.001 | 0.005 | 0.003 | -0.010 | 0.011 | 0.003 | 0.000 |
| **5.CCI=2** | 0.027 | 0.001 | 0.013 | -0.307 | 1.000 | -0.216 | 0.274 | 0.113 | 0.159 | 0.031 | 0.093 | 0.005 | 0.003 | 0.015 | 0.005 | 0.009 | -0.005 | 0.010 | 0.003 | 0.006 |
| **6.CCI=3** | 0.049 | -0.015 | 0.042 | -0.275 | -0.216 | 1.000 | 0.385 | 0.351 | 0.335 | 0.226 | 0.280 | 0.086 | 0.005 | 0.043 | 0.008 | 0.025 | 0.005 | 0.008 | 0.008 | 0.006 |
| **7.Diabetes** | 0.043 | -0.017 | 0.040 | 0.095 | 0.274 | 0.385 | 1.000 | 0.150 | 0.131 | 0.083 | 0.093 | 0.038 | 0.007 | 0.043 | 0.019 | 0.041 | -0.030 | 0.041 | 0.010 | -0.001 |
| **8.CHF** | 0.034 | -0.013 | 0.027 | -0.061 | 0.113 | 0.351 | 0.150 | 1.000 | 0.079 | 0.181 | 0.075 | 0.041 | 0.015 | 0.055 | 0.001 | 0.011 | 0.027 | -0.013 | -0.009 | 0.012 |
| **9.Cerebrovascular** | 0.065 | -0.004 | 0.024 | 0.070 | 0.159 | 0.335 | 0.131 | 0.079 | 1.000 | 0.043 | 0.023 | 0.014 | 0.005 | -0.002 | 0.010 | 0.001 | -0.003 | 0.004 | 0.002 | 0.007 |
| **10.MI** | 0.016 | -0.008 | 0.015 | -0.067 | 0.031 | 0.226 | 0.083 | 0.181 | 0.043 | 1.000 | 0.040 | 0.022 | 0.013 | 0.017 | -0.004 | 0.003 | 0.005 | -0.006 | -0.007 | -0.002 |
| **11.Cancer** | 0.014 | 0.018 | -0.003 | -0.033 | 0.093 | 0.280 | 0.093 | 0.075 | 0.023 | 0.040 | 1.000 | 0.014 | -0.009 | 0.032 | 0.008 | 0.029 | -0.013 | 0.022 | 0.013 | 0.002 |
| **12.Renal diseases** | 0.022 | 0.001 | 0.002 | -0.023 | 0.005 | 0.086 | 0.038 | 0.041 | 0.014 | 0.022 | 0.014 | 1.000 | -0.011 | -0.001 | 0.005 | 0.006 | -0.012 | 0.014 | 0.012 | -0.006 |
| **13.LTC facility beds** | 0.009 | -0.026 | -0.003 | -0.003 | 0.003 | 0.005 | 0.007 | 0.015 | 0.005 | 0.013 | -0.009 | -0.011 | 1.000 | 0.124 | 0.052 | -0.022 | -0.023 | -0.180 | -0.281 | -0.060 |
| **14.social workers** | 0.023 | -0.005 | -0.002 | -0.001 | 0.015 | 0.043 | 0.043 | 0.055 | -0.002 | 0.017 | 0.032 | -0.001 | 0.124 | 1.000 | 0.243 | 0.624 | 0.121 | 0.046 | 0.052 | 0.131 |
| **15.Nurses** | 0.001 | 0.008 | 0.002 | 0.005 | 0.005 | 0.008 | 0.019 | 0.001 | 0.010 | -0.004 | 0.008 | 0.005 | 0.052 | 0.243 | 1.000 | 0.308 | -0.279 | 0.288 | 0.103 | -0.107 |
| **16.Care workers** | 0.000 | 0.003 | 0.009 | 0.003 | 0.009 | 0.025 | 0.041 | 0.011 | 0.001 | 0.003 | 0.029 | 0.006 | -0.022 | 0.624 | 0.308 | 1.000 | -0.304 | 0.466 | 0.191 | 0.092 |
| **17.Living alone** | 0.021 | -0.026 | 0.001 | -0.010 | -0.005 | 0.005 | -0.030 | 0.027 | -0.003 | 0.005 | -0.013 | -0.012 | -0.023 | 0.121 | -0.279 | -0.304 | 1.000 | -0.827 | -0.118 | 0.304 |
| **18.Urbanization** | -0.015 | 0.010 | 0.019 | 0.011 | 0.010 | 0.008 | 0.041 | -0.013 | 0.004 | -0.006 | 0.022 | 0.014 | -0.180 | 0.046 | 0.288 | 0.466 | -0.827 | 1.000 | 0.274 | -0.118 |
| **19.Doctors** | -0.013 | 0.011 | 0.007 | 0.003 | 0.003 | 0.008 | 0.010 | -0.009 | 0.002 | -0.007 | 0.013 | 0.012 | -0.281 | 0.052 | 0.103 | 0.191 | -0.118 | 0.274 | 1.000 | 0.378 |
| **20.Hospital beds** | 0.013 | -0.015 | 0.026 | 0.000 | 0.006 | 0.006 | -0.001 | 0.012 | 0.007 | -0.002 | 0.002 | -0.006 | -0.060 | 0.131 | -0.107 | 0.092 | 0.304 | -0.118 | 0.378 | 1.000 |

Table S3. Cox proportional hazards regression analysis results, using major severe diseases

|  | Crude ratios | | Adjusted ratios | |
| --- | --- | --- | --- | --- |
|  | HR (95% CI) | *p-value* | HR (95% CI) | *p-value* |
| **A. Death or** **Institutionalization** |  |  |  |  |
| Diabetes | 1.226 (1.213, 1.239) | <.0001 | 1.095 (1.082, 1.109) | <.0001 |
| Congestive Heart Failure | 1.514 (1.494, 1.534) | <.0001 | 1.340 (1.319, 1.361) | <.0001 |
| Stroke | 1.186 (1.170, 1.201) | <.0001 | 1.156 (1.140, 1.173) | <.0001 |
| Myocardial infarction | 1.593 (1.553, 1.635) | <.0001 | 1.245 (1.207, 1.284) | <.0001 |
| Cancer | 2.134 (2.103, 2.166) | <.0001 | 1.897 (1.864, 1.931) | <.0001 |
| Renal | 1.806 (1.687, 1.934) | <.0001 | 1.343 (1.234, 1.461) | <.0001 |
| Older adult household | 1.031 (1.021, 1.042) | <.001 | 1.018 (1.010, 1.025) | <.0001 |
| Urbanization | 1.000 (0.997, 1.002) | 0.682 | 0.793 (0.660, 0.952) | 0.013 |
| Number of LTC facility beds | 1.018 (1.014, 1.023) | <.001 | 0.597 (0.393, 0.905) | 0.015 |
| Number of home-visit social workers | 1.559 (1.464, 1.661) | <.001 | 0.993 (0.987, 1.000) | 0.043 |
| Number of home-visit nurses | 0.434 (0.317, 0.594) | <.001 | 1.005 (0.977, 1.033) | 0.740 |
| Number of home-visit care workers | 1.006 (1.003, 1.009) | <.001 | 1.010 (1.003, 1.016) | 0.003 |
| Number of doctors | 0.927 (0.901, 0.954) | <.001 | 0.935 (0.897, 0.975) | 0.002 |
| Number of hospital beds | 1.029 (1.023, 1.035) | <.001 | 1.016 (1.005, 1.027) | 0.005 |
| **B. Death** |  |  |  |  |
| Diabetes | 1.378 (1.362, 1.395) | <.0001 | 1.173 (1.157, 1.190) | <.0001 |
| Congestive Heart Failure | 1.877 (1.851, 1.904) | <.0001 | 1.600 (1.573, 1.627) | <.0001 |
| Stroke | 1.280 (1.262, 1.298) | <.0001 | 1.143 (1.125, 1.162) | <.0001 |
| Myocardial infarction | 1.926 (1.875, 1.977) | <.0001 | 1.351 (1.309, 1.395) | <.0001 |
| Cancer | 3.178 (3.130, 3.226) | <.0001 | 2.578 (2.531, 2.626) | <.0001 |
| Renal | 2.071 (1.930, 2.223) | <.0001 | 1.461 (1.340, 1.594) | <.0001 |
| Older adult household | 1.025 (1.013, 1.036) | <.001 | 1.002 (0.994, 1.010) | 0.661 |
| Urbanization | 1.001 (0.998, 1.003) | 0.639 | 0.946 (0.768, 1.166) | 0.605 |
| Number of LTC facility beds | 1.008 (1.003, 1.013) | 0.002 | 0.571 (0.357, 0.912) | 0.019 |
| Number of home-visit social workers | 2.027 (1.885, 2.179) | <.001 | 0.992 (0.985, 1.000) | 0.038 |
| Number of home-visit nurses | 0.758 (0.533, 1.078) | 0.123 | 1.009 (0.978, 1.041) | 0.566 |
| Number of home-visit care workers | 1.013 (1.009, 1.016) | <.001 | 1.004 (0.997, 1.012) | 0.264 |
| Number of doctors | 0.953 (0.923, 0.983) | 0.003 | 0.927 (0.884, 0.971) | 0.002 |
| Number of hospital beds | 1.011 (1.004, 1.019) | 0.002 | 1.012 (0.999, 1.024) | 0.061 |
| **C. Institutionalization** |  |  |  |  |
| Diabetes | 1.105 (1.089, 1.121) | <.0001 | 1.059 (1.042, 1.077) | <.0001 |
| Congestive Heart Failure | 1.160 (1.137, 1.183) | <.0001 | 1.099 (1.075, 1.125) | <.0001 |
| Stroke | 1.173 (1.152, 1.194) | <.0001 | 1.209 (1.186, 1.232) | <.0001 |
| Myocardial infarction | 1.218 (1.171, 1.267) | <.0001 | 1.133 (1.083, 1.186) | <.0001 |
| Cancer | 1.024 (0.998, 1.051) | 0.075 | 1.017 (0.987, 1.048) | 0.278 |
| Renal | 1.371 (1.233, 1.524) | <.0001 | 1.148 (1.010, 1.305) | 0.035 |
| Older adult household | 1.019 (1.005, 1.033) | 0.007 | 1.030 (1.020, 1.040) | <.0001 |
| Urbanization | 0.998 (0.995, 1.001) | 0.267 | 0.676 (0.528, 0.865) | 0.002 |
| Number of LTC facility beds | 1.025 (1.019, 1.030) | <.001 | 0.543 (0.310, 0.949) | 0.032 |
| Number of home-visit social workers | 0.695 (0.637, 0.759) | <.001 | 0.996 (0.987, 1.005) | 0.340 |
| Number of home-visit nurses | 0.173 (0.112, 0.267) | <.001 | 0.998 (0.963, 1.035) | 0.932 |
| Number of home-visit care workers | 0.988 (0.984, 0.992) | <.001 | 1.015 (1.006, 1.023) | 0.001 |
| Number of doctors | 0.867 (0.833, 0.902) | <.001 | 0.923 (0.873, 0.976) | 0.005 |
| Number of hospital beds | 1.039 (1.030, 1.048) | <.001 | 1.017 (1.002, 1.031) | 0.025 |

CCI: Charlson Comorbidity Index. LTC: Long-Term-Care. HR: Hazard Ratios

Adjusted: Estimation from Multivariate Cox Proportional Hazard Regression analyses for outcomes, using age as the time scale and stratified by sex, residence, disability level, income, health coverage type, and diagnosis year.

Living Alone: The proportion of older adult households where adults aged 65 years or older live alone, per 1,000 households.

Urbanization: The proportion of urban residents per 10 residents in each municipality.

LTC Facility Beds: The number of beds in Long-Term Care facilities (for institutional care) per 100 persons aged 65 years or older.

Home-Visit Social Workers, Home-Visit Nurses, and Home-Visit Care Workers: The number of social workers, nurses, and care workers for home-based care per 100 persons aged 65 years or older.

Doctors and Hospital Beds: The number of doctors and hospital beds per 10,000 persons in each municipality.

Figure S1. Trends in regional LTC resource over time

| Number of LTC facility beds | Number of home-visit social workers |
| --- | --- |
| 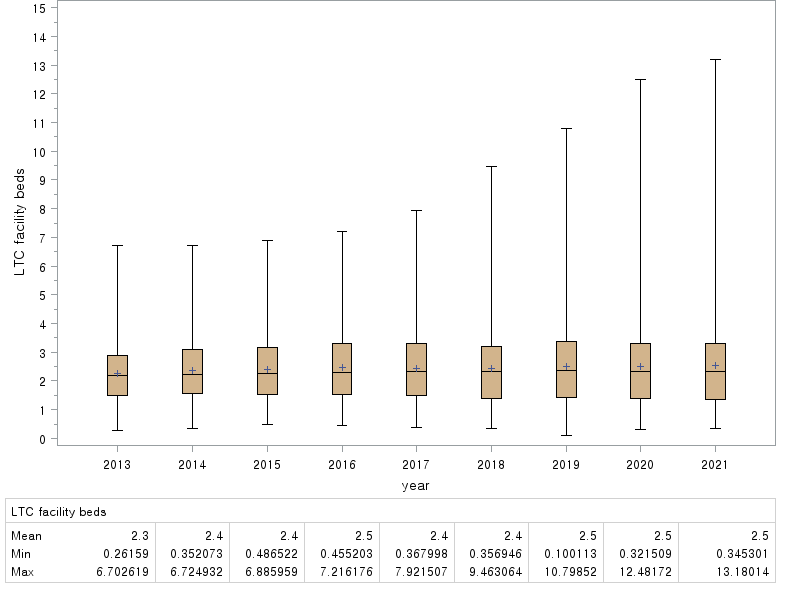 | 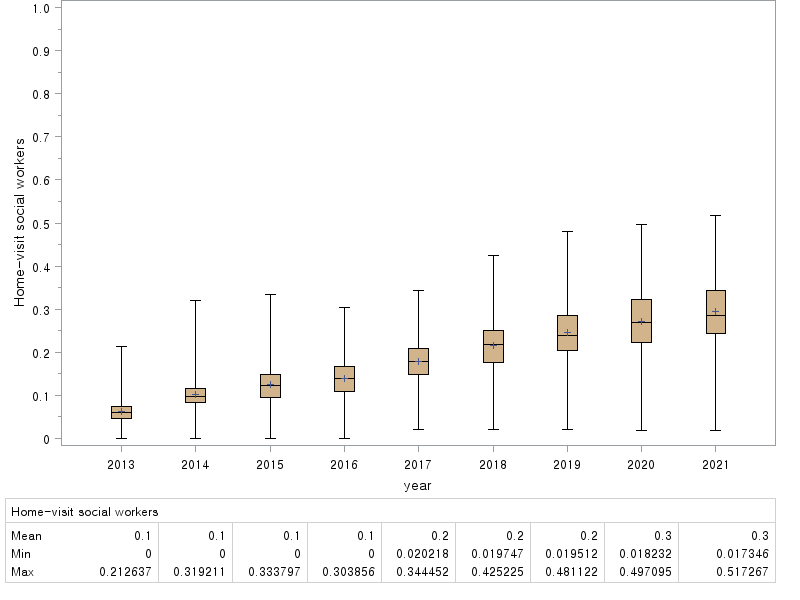 |
| Number of home-visit nurses | Number of home-visit care workers |
| 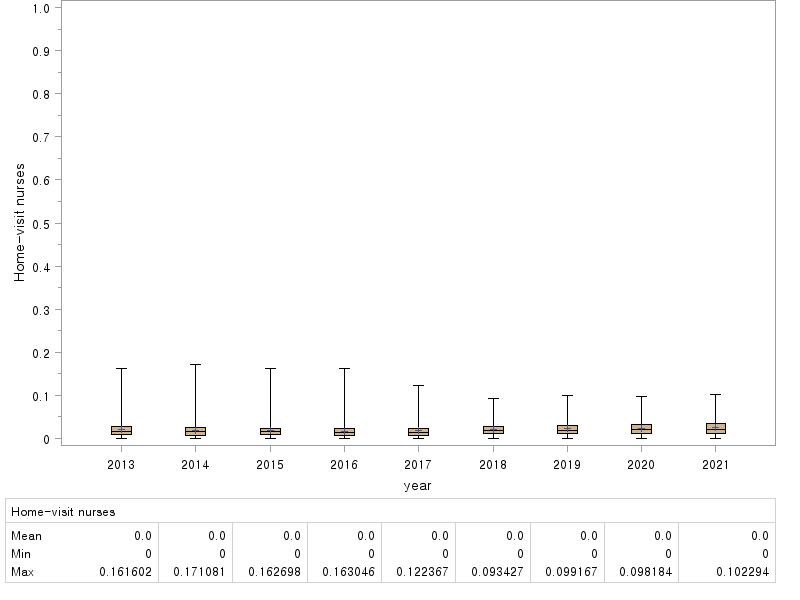 | 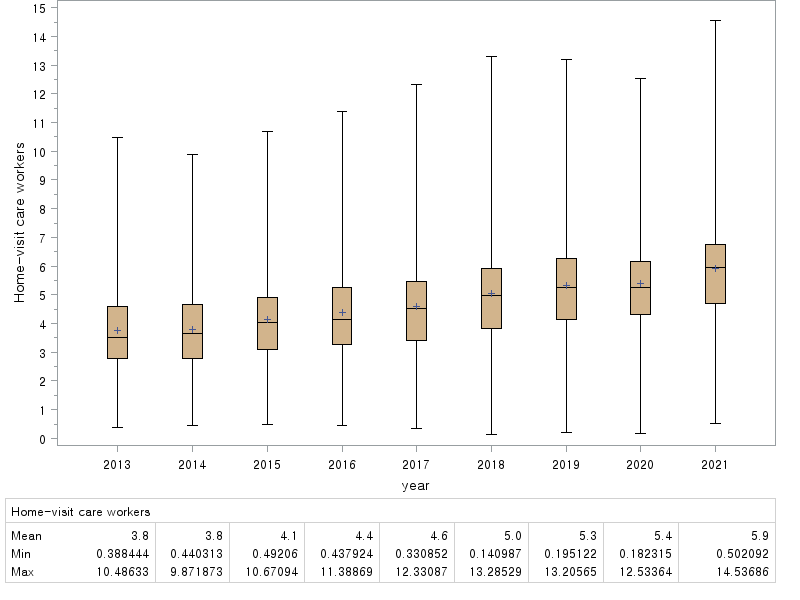 |
| Number of doctors | Number of hospital beds |
| 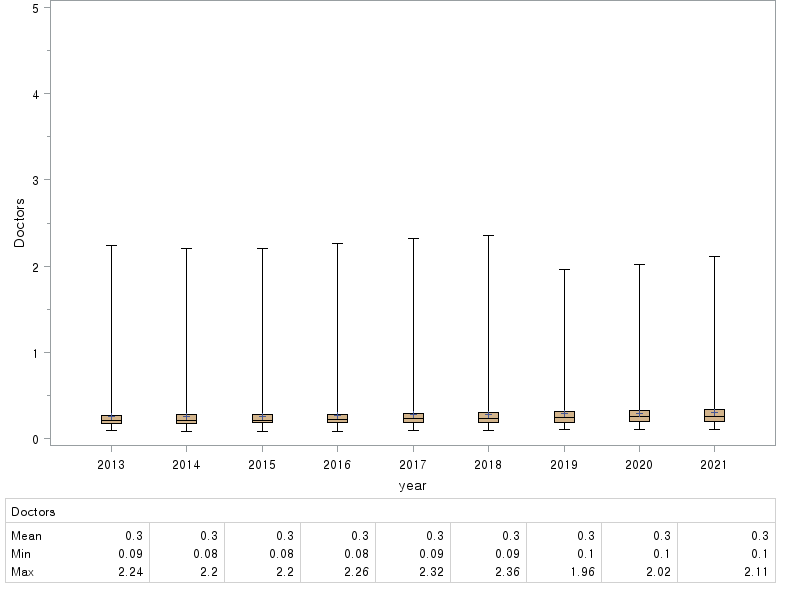 | 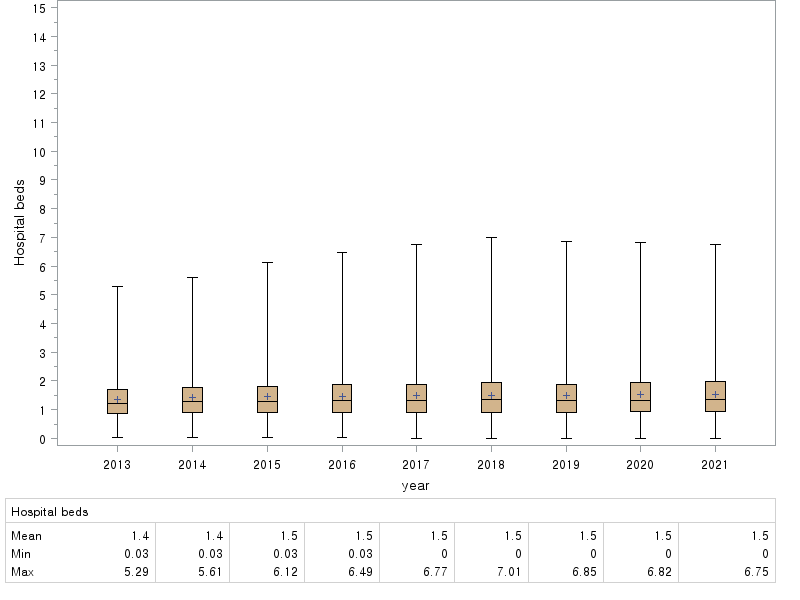 |
